# The Need for Gene Therapy for the Effective Treatment of Hemophilia

**DOI:** 10.4172/2157-7412.S1-016

**Published:** 2012-08-20

**Authors:** Tung Wynn, William B Slayton, Roland W Herzog

**Affiliations:** 1 Department of Pediatrics, Division of Hematology/Oncology, University of Florida, USA; 2 Department of Pediatrics, Division of Microbiology and Molecular Genetics, University of Florida, USA

M.S. is now a 23 year old man who lives in the Caribbean islands. He was born with severe Factor VIII deficiency and at an early age developed Factor VIII inhibitors. He and his family fly from the islands to the U.S. mainland to receive routine hemophilia care since there are no physicians taking care of hemophilia patients and no hemophilia treatment centers on the islands. He has been exposed to various factor products throughout his life including cryoprecipitate, Factor VIII plasma concentrates, and recombinant Factor VIII products. He has also received bypass agents including recombinant activated Factor VII and activated prothrombin complex concentrates, both of which have been very difficult to access. He has acquired Hepatitis C from one or more of his exposures, but remains HIV negative. Despite the presence of inhibitors, he has been unable to undergo immune tolerance induction therapy because of the lack of access to a reliable supply of factor. Because he has been unable to undergo ITI, he has suffered numerous bleeds throughout his life that has left him with hemophilic arthropathy in all of his major joints. He has also had three life-threatening bleeds caused by head injuries that he survived using the limited supplies of bypass agents. He is in chronic pain from his arthritis and has acquired a learning disability from his head bleeds.

This is a tragic case, but unfortunately it is not uncommon for a young man living with hemophilia and inhibitors in 2012.

Hemophilia is an inherited, X-linked disorder that results in abnormal and excessive bleeding in boys and men. It is caused by the low levels or loss of function of the coagulation factor VIII (hemophilia A) and IX (hemophilia B). The frequency and severity of bleeding is dependent upon the degree of residual factor activity. Patients with less than 1% activity have the greatest risk for life or limb threatening bleeds and frequent spontaneous bleeds. These frequent bleeds of the joints result in chronic arthropathy and eventual joint destruction. The incidence of the disorders are 10.5/100,000 males in Hemophilia A and 2.9/100,000 males in Hemophilia B [1]. Patients are usually treated with factor replacing infusions that temporarily correct the deficiency. Because severe patients have spontaneous bleeds, they are treated with factor replacement in a prophylactic fashion, thus preventing bleeds that cause chronic arthropathy. Prophylaxis has been shown to decrease the number of bleeds that patients encounter, improve quality of life, and decrease the overall cost of care [2,3].

Aside from the frequency and severity of bleeds, patients with severe hemophilia also have a higher risk of inhibitor formation. The incidence of inhibitors is 25–30% in severe hemophilia A patients and 3–5% in severe hemophilia B patients [4]. Inhibitors are neutralizing antibodies that develop and lead to the rapid elimination of factor which causes factor infusions to become ineffective. When inhibitors occur patients are at risk for spontaneous bleeds once again and are treated for acute bleeds with bypass agents such as activated factor seven and activated prothrombin complex concentrates. Most patients are also treated with a regimen to induce immune tolerance (ITI). Regular and high doses of factor are given in order to tolerize the immune system and gradually reduce the amount of neutralizing antibodies in order to achieve recoverable levels of factor once again. A typical immune tolerance induction regimen may take a year or more and cost over $1 million. In spite of the cost and effort, up to one third of patients will fail efforts to induce immune tolerance [5]. Quality of life for patients with inhibitors is often poor because they suffer from frequent bleeds. The bypass agents, although usually effective, do not prevent bleeds and add cost to an already extremely expensive problem while exposing patients to viral infections and the potential of Creutzfeldt-Jakob disease. Patients undergoing immune tolerance induction require the frequent dosing of factor and require frequent follow-up at a centers with skill and expertise in the treatment of hemophilia and inhibitors. Furthermore, patients with factor IX inhibitors have been reported to develop severe, sometimes life-threatening immune reactions to factor during immune tolerance induction making completion of the ITI nearly impossible [6].

Gene therapy can transfer a functional gene to patients with congenital factor deficiency in order to produce the factor in high enough quantities and in a sustainable fashion. Most frequently, the genes are transferred using a viral vector, with lentivirus vectors and adeno-associated virus (AAV) vectors being the most common. This type of gene transfer has shown the most clinical success, but limitations include the finite amount of gene constructs that can be carried, incomplete expression of gene products, immune destruction of viral antigens, the risk of insertional mutagenesis (with lentivirus, in particular), and the eventual loss of gene expression.

These limitations are quickly being overcome and the potential of gene therapy is being demonstrated. First, in animal models primarily utilizing factor IX, AAV has been demonstrated to be an efficient vector in both mice models and hemophilia B dog models [7,8]. In addition, factor IX deficient mice largely have remained free of inhibitors when gene transfer occurred within the liver [9]. The degree of tolerization in these mice was dependent upon the degree of protein expression. Most recently an international collaboration reported stunning success. The team treated 6 patients with severe Factor IX deficiency using an AAV8 vector. All patients were able to show expression of Factor IX after liver directed gene transfer in a dose dependent fashion. The expression has been sustained for up to 22 months and allowed all patients to either receive factor less frequently or to be able to stop prophylaxis altogether [10,11].

## Conclusion

Gene therapy is promising to patients with hemophilia, with or without inhibitors. It offers the promise of life without the risk of bleeding after a simple injury and chronic arthropathy. It provides the promise of life without IV infusions for factor replacement. It makes the promise that no person with hemophilia will have to live a tragic life. Restoring a patient’s ability to make a protein that was congenitally deficient and all but curing the disorder is the goal of everyone treating any heritable disease and is the goal of gene therapy for hemophilia patients. Ultimately, gene therapy promises life without hemophilia. The goal and promise of gene therapy is well worth striving for.
